# Spatio-Temporal Dynamics of Maize Yield Water Constraints under Climate Change in Spain

**DOI:** 10.1371/journal.pone.0098220

**Published:** 2014-05-30

**Authors:** Rosana Ferrero, Mauricio Lima, Jose Luis Gonzalez-Andujar

**Affiliations:** 1 Departamento Protección de Cultivos, Instituto de Agricultura Sostenible, Consejo Superior de Investigaciones Científicos (CSIC), Córdoba, Spain; 2 Departamento de Ecología, Pontificia Universidad Católica de Chile, Santiago, Chile; 3 Laboratorio Internacional de Cambio Global, LINCG (CSIC-PUC), Santiago, Chile; 4 Center of Applied Ecology and Sustainability (CAPES), Santiago, Chile; Institute for Sustainable Agriculture (IAS-CSIC), Spain

## Abstract

Many studies have analyzed the impact of climate change on crop productivity, but comparing the performance of water management systems has rarely been explored. Because water supply and crop demand in agro-systems may be affected by global climate change in shaping the spatial patterns of agricultural production, we should evaluate how and where irrigation practices are effective in mitigating climate change effects. Here we have constructed simple, general models, based on biological mechanisms and a theoretical framework, which could be useful in explaining and predicting crop productivity dynamics. We have studied maize in irrigated and rain-fed systems at a provincial scale, from 1996 to 2009 in Spain, one of the most prominent “hot-spots” in future climate change projections. Our new approach allowed us to: (1) evaluate new structural properties such as the stability of crop yield dynamics, (2) detect nonlinear responses to climate change (thresholds and discontinuities), challenging the usual linear way of thinking, and (3) examine spatial patterns of yield losses due to water constraints and identify clusters of provinces that have been negatively affected by warming. We have reduced the uncertainty associated with climate change impacts on maize productivity by improving the understanding of the relative contributions of individual factors and providing a better spatial comprehension of the key processes. We have identified water stress and water management systems as being key causes of the yield gap, and detected vulnerable regions where efforts in research and policy should be prioritized in order to increase maize productivity.

## Introduction

Spatio-temporal patterns of agricultural production are clearly influenced both by climate change and agricultural management practices. Recently, many studies have analyzed the impact of climate change on crop productivity [1], but comparing the performance of different crop management systems has rarely been explored (exc. [2]). To be specific, we need to evaluate how and where irrigation practices (e.g. rain-fed versus irrigated) are effective in mitigating the effects of climate change, because water constraints and crop demand in agro-systems could be increased due to climate change [3]–[7]. Identifying whether there are any differences in the principal bio-physical factors and mechanisms that explain both systems will enable us to improve crop productivity without expanding the cropland area and to diminish the adverse impacts of agriculture for social and ecological systems [8].

We do not know much about crop response to climate change yet, and still less about the differential response between irrigated and rain-fed systems [4]. Increases in agriculture production could potentially come from increases in irrigated crops, because higher yields could be attained with reduced production variability [9]. However, this also depends on soil and management factors that result in spatial patterns of yields [10]. Secondly, irrigation can influence local climate by inducing cooling, but this may depend on the extent of the irrigated area, the level of soil moisture alteration and cloud response to irrigation [11]. Third, average yields in rain-fed systems are commonly 50% or less of yield potential (high yield gap), suggesting ample room for improvement [12] but, again a great spatial variability has been found [13]. Yield gaps could be bigger in cropping systems that experience wider ranges of variation under climate conditions [10]. Fourth, plant population (or density) is known to affect the yield potential at a given location [12] and grain yield stability [14]. However, to our knowledge, there are no previous studies explicitly comparing endogenous processes under different water management systems. Finally, simulation at a broad scale level cannot fully explain the above process, and process-based crop models do not always relate to observed yields [15]. Finer spatial scales and historical data of irrigated versus rain-fed systems could help to compare modelled or simulated yield potentials [12].

Analyzing the sensitivity of irrigated and non-irrigated (rain-fed) crops to past climate changes is crucial to an understanding of the vulnerability of agriculture to climate change in the future, particularly in regions that already suffer from this under present conditions. This paper explores biophysical factors and water management practice constraints to maize (*Zea mays* L.) in Spain. Spatial shifts northwards have been projected for maize, due to the extremely hot, dry summers in south-central Europe [16], [17], particularly in Spain [18]. The expected effects of climate change on Spain's agriculture would not be uniform. Mediterranean (arid and semiarid) regions may be particularly sensitive, where a decrease in the general availability of hydric resources and an increase in evaporative demand, especially during summer, will affect irrigation requirements [19]. Namely, it is one of the most prominent “hot-spots” in future climate change projections [20], where a mean reduction of 17% in water resources [21], [22] has been predicted. For this drought-prone zone, all climate change scenarios imply the need to significantly increase the contribution of irrigation water. Therefore, identifying and quantifying the links between water management practices and food production is crucial in addressing the intensified conflicts between water scarcity and food safety.

The objective of this paper is to determine how climate variability affects maize production in Spain under irrigated and rain-fed conditions. First, we have analyzed the regulatory structure of maize production dynamics under both water management systems. Second, we have evaluated the mechanisms (in ecological parameters) underlying climate perturbations on maize yields. Third, we have assessed whether the importance of maize production structures (i.e. intrinsic regulation) and climate change perturbations (i.e. exogenous factors) could change according to the type of management (i.e. rain-fed and irrigated) and the geographical location. Fourth, we have estimated the potential yield of each region and water management using the previous models and analyzing the spatial variability of yield losses due to water stress [23]. We have combined information on spatial autocorrelation water stress patterns for maize yields to identify the importance of climate constraints at a regional scale.

## Methods and Materials

### Database

**Figure 1:**
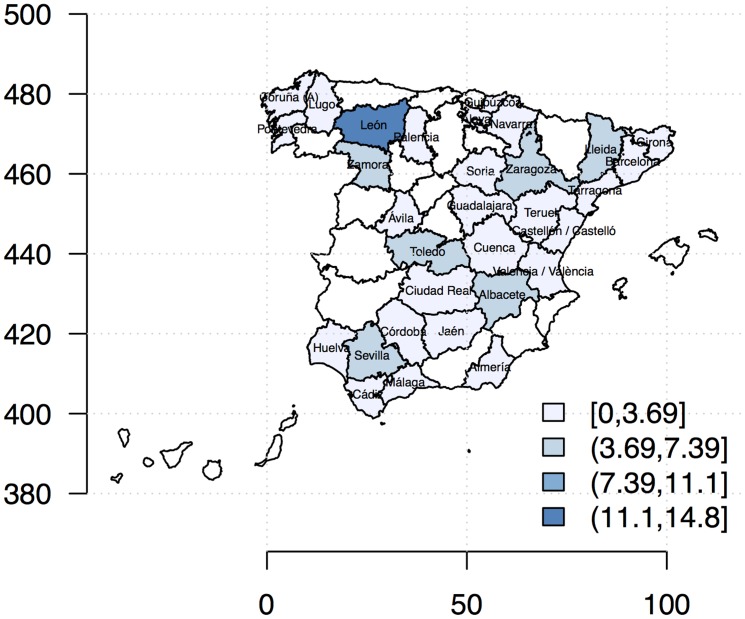
Definition of study regions (provinces) with percentage of total maize production for 1996–2009. Only provinces with both irrigated and rain-fed systems were analyzed.

Provincial maize yield levels (*Zea mays*; production per hectare, *kg*/*ha*) for 1996–2009 were obtained from statistical yearbooks [24]. We studied selected provinces that had both rain-fed and irrigated systems (Fig. 1), and displayed trends in yield fluctuation in Fig. S1. We used Global Historical Climatology Network (GHCND) data on monthly temperature and rainfall (mean, minimum, maximum and extreme; [25]). Various summary statistics of the growing season (July to October) weather were then computed: *EMNT* extreme minimum temperature (°C), *EMXT* extreme maximum temperature (°C), *MMNT* mean minimum temperature (°C), *MMXT* mean maximum temperature (°C), *MNTM* mean temperature (°C), *EMXP* extreme maximum daily precipitation total (l/m2) and *TPCP* total precipitation (l/m2). We also examined carbon dioxide emission (*CO_2_*), an important atmospheric gas that contributes to global warming. The annual country-level emissions of *CO_2_* (*kt*) were taken from the World Bank's World Development Indicators (WDI; [26]).

### Diagnosis and statistical models of yields dynamics

We have analyzed and predicted maize yield responses to the impact of climate change in Spain through the use of models based on the population dynamics theory. Of course this is not a true population in the reproductive sense, but crop systems obey the same rules as all other dynamic systems, both natural and engineered.

First, where necessary, we used sequencing (i.e., splitting the series into two stationary segments) and detrending (i.e., rotating the series around the linear or quadratic trend) to generate a stationary time series. Second, we estimated the logarithmic rate of change of the yield as 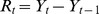 (the same response variable as [1], [27]), where 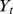 represents the provincial yield in a year 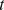
(the logarithm of the detrended yield) and 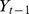 is the same series with one year of delay (lag 1).

We were able to detect and analyze non-trivial feedback processes by examining their relationship 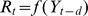, where the function 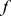 described how the crop yield change rate varied with yield level, and this has been called the *R*-function. We used the partial rate correlation function (or *PRCF*) to estimate the order of the dynamical process and determine how many time lags (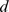) should be included in the model for representing the feedback structure. This function detects the feedback order removing the confounding effect by calculating the partial correlation between 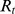 and 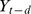 with the effects of lower lags removed [28].

We then used the generalized version of the exponential form of the discrete time logistic model [29], [30] in terms of the *R*-function to represent pure endogenous models in the function 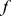:
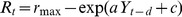
(1)where 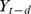 represents the yield data at time 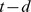 (where 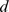 was obtained from *PRCF* function), 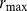 is a positive constant representing the maximum finite rate of change (and is estimated as the maximum rate of change from the observed data), 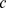 is a measure of the ratio between demand and offer of limiting resources and 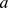 is the nonlinearity of the curve. The nonlinearity of this model includes a biological realistic property: its net reproductive rate is bounded [29], that is, the performance of any crop must have an upper bound simply because no crop can produce an infinite number of grains that subsequently contribute to the crop yield.

Finally, we used the Royama classification of exogenous effects as a framework to deduce causal mechanisms of the climate change impact on these crop yields in a spatial-temporal study [29]. To include exogenous perturbations, we modelled 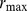 and 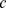 of (1) as linear functions of climate conditions, each of which has an explicit biological interpretation. In this way, we set up mechanistic hypotheses about the exogenous effects of climate on these yields data [29].

If an exogenous factor (i.e. climate or gas emissions) changes 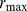 and has an additive or independent perturbation effect on crop yield levels, it shifts the *R*–function curve along the *y*-axis (“*vertical*” perturbations):
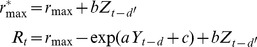
(2)where 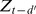 is the exogenous factor (for lags or 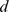 0 and 1; in logarithm scale). This model produces alterations to both 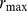 and the carrying capacity (equilibrium point of the population, 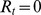), changing the level of equilibrium and its stability.

If an exogenous factor (i.e. climate or gas emissions) changes *c* and has a non-additive perturbation effect on crop yield levels, and influences the equilibrium point of the population shifting the *R*-function curve along the *x*-axis (”*lateral*” perturbations):
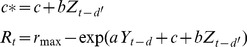
(3)Lateral perturbations do not change the pattern of dynamics around equilibrium because they do not change the slope at the equilibrium.

We fitted Eqs. 1–3 using nonlinear least squares regressions with the *nls* library in the software *R* [31], [32]. In particular, the models were fitted by minimizing the Akaike criterion with a correction for finite sample sized (*AIC_c_*):
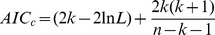
where 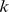 is the number of parameters and 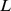 is the maximized value of the likelihood function for the model, and 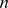 denotes the sample size. Also, we maximized the pseudo *R^2^* measures based on the deviance residual [33]. Models were chosen on the basis of their goodness-of-fit (assessed using root mean square error RMSE and the log-likelihood values), their ability to describe the correct feedback structure, and their appropriateness.

### Yield losses due to suboptimal water availability (YGRw)

We propose a new estimation of the potential yield or equilibrium productivity [34] at the provincial level as the equilibrium value of the models. By solving Eqn. (1–3) for the equilibrium dynamics 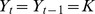 (when 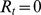), we calculated the maize yield level at equilibrium, sometimes called the carrying capacity (*Mg/ha*). For non-pure endogenous models we made potential yield estimations for each year as the exogenous factor changed. Then we calculated the percentage of yield losses due to suboptimal water availability (*YGRw*; Eqn. 4; view [35]), which indicated how close the rain-fed yield potential is to the irrigated value for a given site (%).
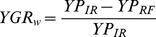
(4)We obtained some time-invariant *YGR_w_* values when, in the same province, irrigated and rain-fed *YP* were estimated from pure endogenous models, so that we calculated the averaged *YGR_w_* for each province, and studied its spatial variability without taking into account the temporal dimension of the data.

We determined whether there was any spatial autocorrelation in *YGR_w_* with the global Moran's *I* (spatial correlation on average, of an entire map). At this stage, we were not yet trying to determine the causes, although the results could have motivated a hypothesis. We assumed: 1) that there was no spatial patterning due to some underlying but unmodelled factor, and 2) that the assigned spatial weights were those that generated the autocorrelation. Then we tested whether *YGR_w_* was more spatially clustered than by chance. The matrix that represents spatial dependence (*W*) uses a binary indicator of neighbourhood (i.e. the spatial weights, *w_ij_*, are defined as *w_ij_* = 1 if the *i* and *j* provinces are contiguous neighbours, *w_ij_* = 0 otherwise, based on rook contiguity; [36]). We used row-standardisation (style *W*) that favours observations with few neighbours. We calculated a non-parametric approach to inference on Moran's *I* using 999 simulations (Monte Carlo permutation test). Also, local indicators of spatial association (or LISA) were calculated to detect “hot spots” where there was a strong autocorrelation, and “cold spots”, where there were none. The results were plotted on a Moran scatterplot: the target variable on the *x*-axis, and the (spatially-weighted) sum of neighbouring values on the *y*-axis; these are called spatially lagged values. We identified the high-influence areas.

We analyzed the environmental spatially distributed causes of averaged *YGR_w_* through a Simultaneous Autoregressive Model (SAR; [36]) that considers spatial autocorrelation of residuals:
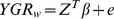
(5)where, for each province, *YGR_w_* is the percentage of yield losses due to suboptimal water availability, 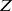
is a matrix of averaged climate variables (see Database section; except country-level *CO_2_* emissions), 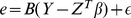 is the error term, and 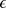 represents residual errors (assumed to be independently distributed according to a Normal distribution with zero mean and diagonal covariance matrix 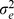). The error terms are modelled so that they depend on each of the other areas to account for their spatial dependence (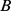 is a matrix that contains the dependence parameters; 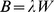, where 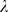 is a spatial autocorrelation parameter and 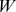 is a matrix that represents the spatial dependence explained above). Global Moran's *I* was computed for the residuals to test if the SAR model accounts for all the spatial autocorrelations in *YGR_w_*. For the spatial analysis we used *spdep* library in the software *R* [37].

## Results

### Regulatory structure and exogenous perturbation models

After sequencing and detrending, all the sites exhibited first-order negative feedback (*PRCF(1)*) as being the most important component of yield growth rate (Figure S2; except for irrigated maize in Vizcaya and rain-fed systems in Tarragona). Major sites showed the highly significant (*p*<0.05) effect of endogenous processes as determinants of the structure of crop productivity regulation (Table S1).

**Figure 2:**
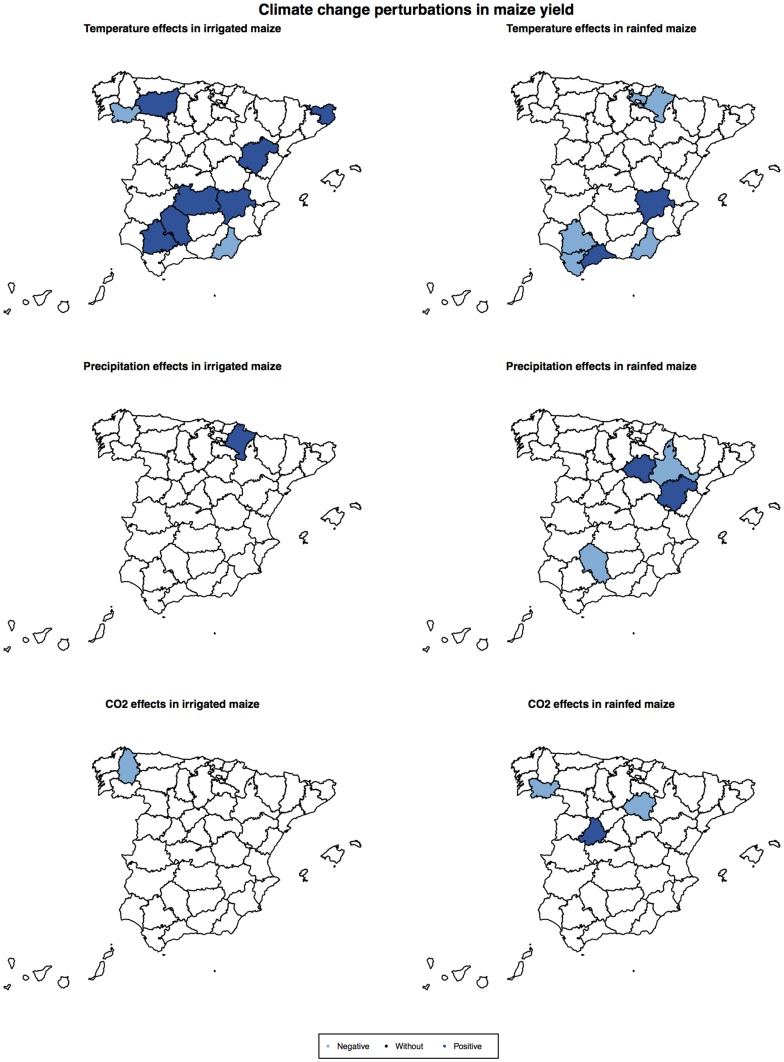
Effects of temperature, precipitation and CO2 emission, on maize productivity for rain-fed and irrigated crops. Provinces for both water management systems were selected for the analysis. All models are from Table S1.

We evaluated gas emission (*CO_2_*) and climate factors (temperature and precipitation; see Database section), as exogenous perturbations of the production curve (*R*-function). Table S1 shows several models that were selected as climate change impacts on maize production for each Spanish region and management system. The stochastic versions of the step-ahead predictions of the models are shown in Fig. S3. As expected, the effects of climate on maize production were not uniform, and depended on the irrigation management system (Figure 2). Maize yields were significantly related to minimum temperatures (possibly night ones) in 11 sites and by maximum temperature in another 5 sites. Generally, there were positive effects of temperature for irrigated systems, except for Almería (for minimum temperature –*EMNT*-) and Ourense (mean temperature –*MNTM*-). However, for rain-fed systems, we detected negative effects of warming on major sites, with the exception of Málaga and Albacete (both for *EMNT*). As expected, precipitation was not important for irrigated systems (except for maximum precipitation –*EMXP*- in Navarra), but it was an important factor in some rain-fed managements. There were positive effects of precipitation on Teruel and Soria (for a total –*TPCP*- and maximum rainfall), and negative ones in Córdoba (*TPCP* and *EMXP*) and Zaragoza (*TPCP*) on rain-fed crops. Finally, *CO_2_* emissions negatively affected maize in Lugo (irrigated), Ourense and Soria (both rain-fed), and positively only in Ávila (rain-fed).

**Figure 3:**
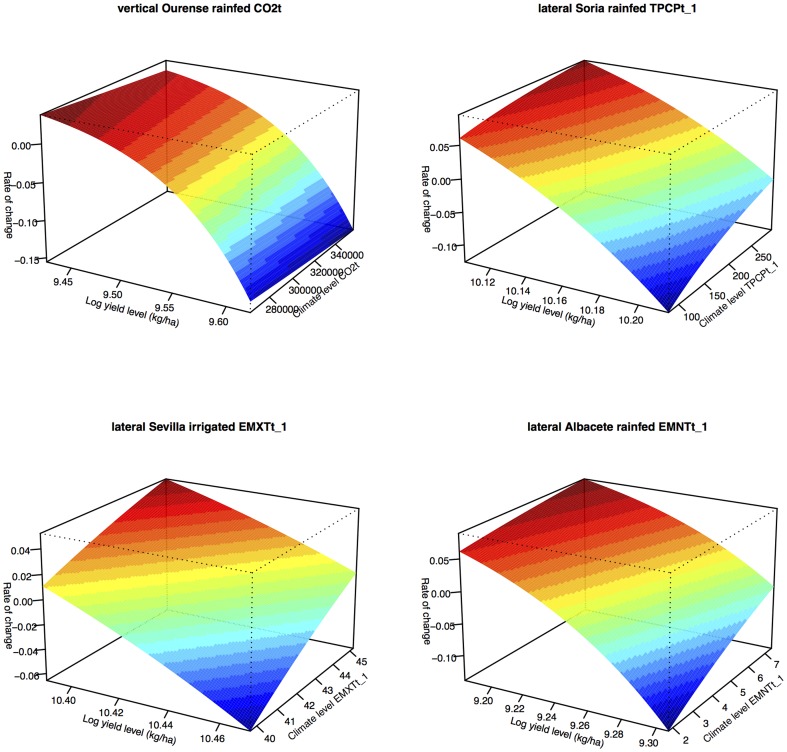
Yield rate of change against the log observed yield level (with one year of delay) and the exogenous factor that perturbs the productivity function (*R*-function). Exogenous factors include carbon emissions (*CO2_t_*), precipitation (*TPCP_t_1_*), and maximum and minimum temperature (*EMXT_t_1_* and *EMNT_t_1_*). Additive (vertical) and non-additive (lateral) perturbation effects were detected. Colours indicate the *R*-function value. See Table S1 for description of models and Figure S4 for their graphs.

Temperature acted mainly as having non-additive (lateral) effects on maize yield dynamics, whereas *CO_2_* emission acted as additive (vertical) effects (Table S1; Figure 3 and S4). Finally, rainfall exerted non-additive effects when it had a negative impact on maize, but when it obtained positive responses the effects were of both types (additive or non-additive; Table S1; Figure S4). For example, Figure 3 shows positive and non-additive (lateral) effects of temperature on rain-fed maize in Albacete and irrigated maize in Sevilla. That is, the increase in temperature had a positive effect on both maize systems, and more so at high yield levels. Figure 3 also indicates a negative and additive (vertical) effect of *CO_2_* emission on Ourense (same strength for all yield levels), and a positive and non-additive (lateral) rainfall effect on rain-fed maize in Soria (more important for high yield levels).

### Relative yield losses due to suboptimal water availability (YGR_w_)

**Figure 4:**
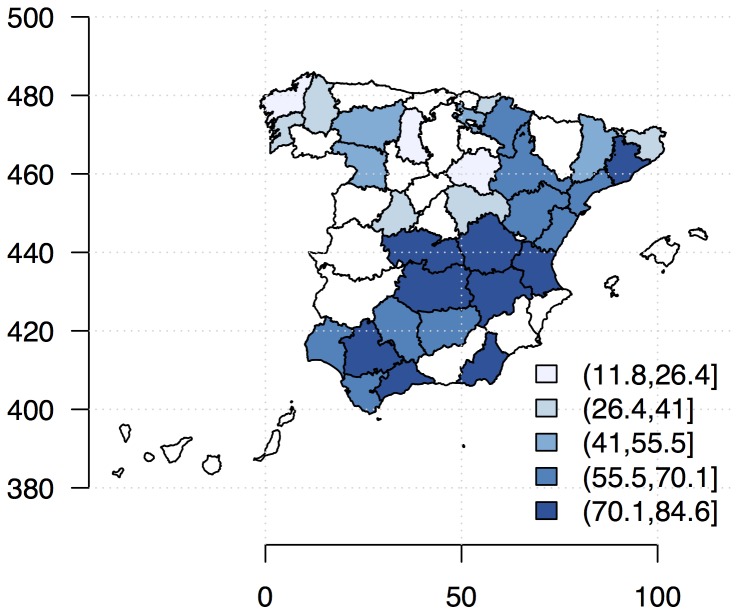
Relative yield losses due to suboptimal water availability (*YGRw*; *%*). The percentage of yield losses due to suboptimal water availability indicates how close rain-fed yield potential are to the irrigated value for a given site.

**Figure 5:**
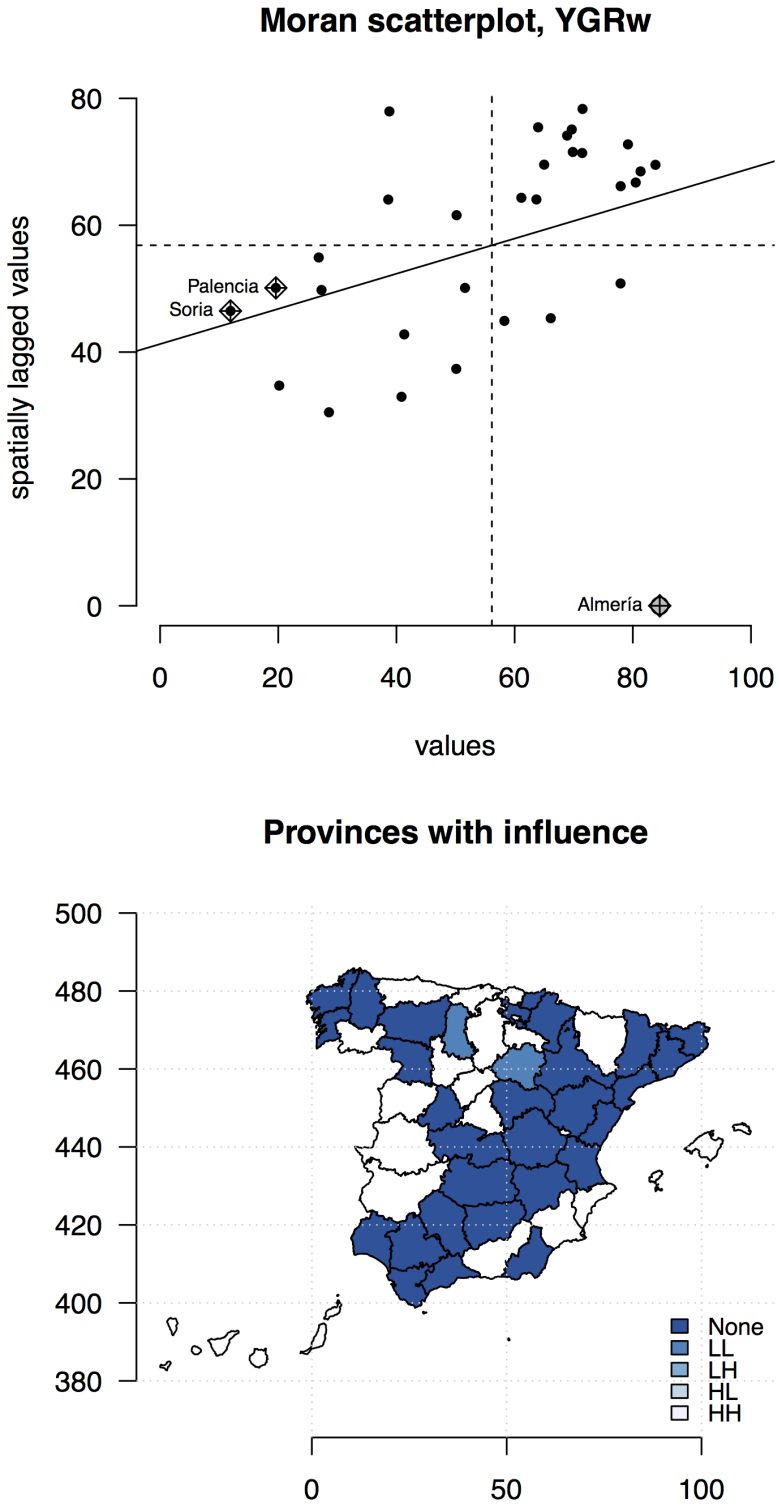
Spatial autocorrelation analysis of the relative yield losses due to suboptimal water availability (*YGRw*). Top: Moran scatterplot; bottom: high-influence areas neighbours: no influence (*None*), high proportion with low proportion neighbours (*HL*), the reverse (*LH*), and both high (*HH*). We define the break between “low” and “high” as the third quartile.

We first visualized the spatial relation of *YGR_w_* (Figure 4), where several high *YGR_w_* values were shown in central and southern provinces of Spain. The global Moran's *I* value (*I* = 0.39) was of an opposite sign and much larger in absolute value than the expectation (E[*I*] = −0.034); this was quite unlikely to be equal to the expectation of no spatial association. The probability of incorrectly rejecting the null hypothesis of no association (type I error) was 0.0021. The Monte Carlo approach also rejects the null hypothesis (the true value for Moran's *I* is zero; *I_mc_* = 0.406, *p* = 0.005; Figure S5). The Moran scatterplot (Figure 5; the vector of values and the neighbour list with weights) showed points with a great influence, which are identified by a special symbol and their name. The highest-leverage area is marked on Almería; it has the highest *YGR_w_* (84.56) and a zero weighted spatially-lagged proportion, because it did not have any adjacent areas in the study. Soria and Palencia had low *YGR_w_*, and a low spatially-lagged proportion; these are the low-*YGR_w_* neighbourhoods adjacent to low-*YGR_w_* neighbourhoods. They have a great influence on the slope (global Moran's *I*). From Figure 5 it is clear that most of the global Moran's *I* significance comes from the local Moran's *I* from high *YGR_w_* in Almería, and low *YGR_w_* associated with low *YGR_w_*, in the Soria and Palencia area in the north.

There was clear evidence of local clustering, 6 areas (Ciudad Real, Cuenca, Albacete, Valencia, A Coruña and Pontevedra) showed sufficiently high local Moran's *I* to reject the null hypothesis with less than a 5% chance of Type I error. These areas were not highlighted in the Moran scatterplot, as they did not greatly influence the global Moran's *I* but were locally-clustered.

There was a significant spatial correlation in the residuals, because the estimated value of lambda was 0.141 and the *p*-value of the likelihood ratio test 0.0354. Only averaged temperature (*MNTMt*) was significant for the SAR model, suggesting that provinces with higher temperature have larger *YGR_w_* percentages. The model found was: 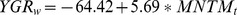 the SAR model, which accounted for the whole spatial autocorrelation in *YGR_w_* (global Moran's test for residuals was *I* = −0.00811, *p* = 0.422). Thus, the autocorrelation in the linear model residuals was explained.

## Discussion

In the present study, the impact of climate variability on maize yields in Spanish rain-fed and irrigated systems was investigated for the period 1996–2009. We explored the endogenous structure (regulation) and the exogenous perturbations effects on maize production at a regional scale.

### Regulatory structure: endogenous feedback

We found that maize productivity had a persistently negative effect on crop yields for a one year time delay (first order negative feedback, *PRCF(1)*). Maize productivity was characterized by negative first-order feedback structure in major sites and in both irrigation systems. Namely, there were biomass or density-induced feedback loops in the growth, survival rates, seed germination or grain production rates of individual plants, tending to stabilize their dynamics [38]. In Spain, the seeds produced are used for the next year and, therefore, a year's crop performance could change seed viability and vigour, which also affects the performance of the following crops (changing the demand for resources). Also, a crop system could alter habitat conditions; in fact, the frequent practice of crop rotation is a testimony to the importance of negative feedbacks in agricultural systems (i.e. it modifies resource supplies). This produces high-frequency dynamics due to year-to-year endogenous variability in maize yields. Our logistic models appear to capture the essential features of the fluctuations observed, and suggest a mechanistic explanation for the latter. This implies that, to understand the response of maize productivity to climate, we must also know the endogenous feedback structure of the system.

Our models are important to conceptualizing the problem of regulated versus unregulated systems. If a system were to be controlled entirely by an exogenous process (unregulated systems), then the series would perform a random walk and we saw no sign of the generated series becoming stabilized, but it drifted increasingly away from the origin with the passing of time [29]. However, persistence implies regulation (but not necessarily vice versa) and, therefore, the rate of change in a persistent crop productivity system is not statistically independent of the yield level and should be bounded (i.e. regulated systems).

### Climate change effects: exogenous perturbations

In line with previous studies, temperature during the growing season was the most important weather variable influencing maize yields [39]. However, we deciphered the effects of climate on maize productivity providing new interpretations. First, diagnostic analysis suggested that temperature acts mainly as a non-additive (lateral) perturbation in maize productivity. Therefore, the relationship between temperature and maize yields was nonlinear and could not be captured adequately by a linear or quadratic functional relation as in previous studies [40]. Our analysis suggests a biological reason for the nonlinear interaction between climate and maize yield level. Temperature had no direct impacts on yield rate of change (affecting 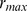; additive or vertical effects), but influenced the availability or requirements of some limiting factor or resource (changing 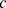; non-additive or lateral effects). There is probably a relationship between extreme heat and plant water stress, increasing water demand and/or soil water content in rain-fed systems, in agreement with the recent results of Lobell *et al*. [41]. This is because, the effects of high temperature are experienced only when the maize yield level is close to equilibrium [29]. This kind of perturbation exerts strong effects on the average level of yield but few on the intrinsic periodicity induced by endogenous feedback.

Secondly, rain-fed maize yields are negatively affected by temperature increases, but irrigated systems may gain from warming in some regions. As expected, rain-fed crop damage may result from greater water and heat stress during hot growing seasons. However, unexpected positive effects of temperature in irrigated systems are possibly a consequence of heat tolerance, which is consistent with other studies on local adaptation to hot temperatures being able to minimize stress effects [40] or the cooling effect of irrigation [42]. Therefore, we detected some adaptation to heat stress that could mitigate the projected heat-related losses, at least in a few regions with irrigated systems.

Thirdly, climate variability and extreme events are more important than averages. Thus, we detected that minimum temperature was the dominant factor in maize production, in agreement with other recent studies for maize [43]–[45] and rice [46]. Currently, a new paradigm has been originated: crop yields have declined with a higher minimum or night temperature [46], [47] or when there was a marked asymmetry between maxima and minima [48]. One possible explanation includes the facts that the grain-growth rate has increased and that the duration of grain-filling has been shortened as the temperature increased, producing lower crop production (yield levels) [49]. Mohammed & Tarpley [47] proposes a list of the effects of high night temperatures on crop production. Also, our findings are in line with the results of recent research which argue that global minimum temperatures are increasing faster than maximum temperatures, and the need to explore the ecological consequences of this phenomenon [41], [50], [51]. Therefore, we wish to highlight the importance of considering extreme climate variables in crop production studies, and limiting the use of averages or accumulative climate data which ignore inter-annual variability of climate and extreme events. Our results differ from those of most studies which do not take into account food production structure regulation, and those which use degree-days [40] concepts which assume a cumulative or additive effect of temperature on crop yield and do not adequately account for the effects of extreme temperatures (high or low) either.

In the study period, precipitation was not a major abiotic factor limiting maize yield of cultivated rain-fed crops in Spain. We only detected positive effects of precipitation for irrigated maize in Navarra, Teruel and Soria. Also, growing season rainfall negatively affected rain-fed maize yield in Córdoba and Zaragoza, possibly due to flood and waterlogging problems causing production losses. Again, we agree with Lobell *et al*. [41], who argue that the apparent paradox of the scant effect of precipitation on rain-fed maize yield whereas, on the contrary, there is a water stress effect of temperature, can be solved with the following reflection “*large precipitation changes are required to rival the effect of temperature on water stress, because high temperature affects both water demand and supply*”.

As in the study of Long *et al*. 2006 [52], ours study indicates that there was a smaller *CO_2_* effect on maize yield than previously presumed. Impacts of higher *CO_2_* on maize yield were reduced probably because it is a C4 plant, and also because of the national scale of the variable in our study.

### Spatial variability of yield losses due to water stress

We found that the global spatial pattern of yield losses due to water stress is not a random one (Figure 4); there was a high influence in Palencia, Soria (lowest) and Almería (highest). We detected clusters of “cold spots” in northern Spain (A Coruña and Pontevedra) and “hot spots” in central provinces (Ciudad Real, Cuenca, Albacete, Valencia; Figure 5). Neither cluster greatly influenced the global Moran's *I* but they were locally-clustered. Moreover, we modelled spatial *YGR_w_* values with climate variables and found that the mean temperature was the highest constraint of maize productivity due to water stress. In conclusion, policy action to decrease the relative yield gap due to water stress on maize productivity has the potential to geographically target high *YGR_w_* areas. Future work will help determine other non-climatic causal relationships between *YGR_w_* and an array of factors that could influence water management practices in maize (e.g. access to water, management technology, soil conditions, etc.).

A recent comparison of simulated and observed yield patterns highlights the value of data in the spatial distribution of yields for understanding the causes of landscape yield variability [10]. However, to our knowledge, this is the first study explicitly evaluating the spatial pattern of real relative yield gaps due to a water management system and its sensitivity to underlying climate factors. The results demonstrate that spatial patterns of yield loss due to water stress possess substantial information on the relative importance of water management factors for maize productivity.

The need for an analysis to identify and implement adaptation options in agriculture emphasizes the importance of regional scales (federal, provincial, and territorial governments). Global and non-spatial studies can provide only a very partial and potentially misleading insight into the true impact of climate change, where aggregation can indeed conceal vulnerability and climate change costs [53]. However, individual regions (provinces) allow a better analysis of uncertainty and risks, thus providing practical recommendations to farmers.

## Conclusions

We identified the same regulation structure for both management systems, i.e. a negative first-order feedback process that tends to stabilize the crop's dynamics. We analyzed the underlying mechanisms of the interaction between climate variation and regulatory structure on maize production. Different climate variables appear to operate differently on maize productivity. We found that the effect of temperature (mainly extreme values) cannot be evaluated independently of crop productivity as in previous studies, because its consequences are experienced only when maize yield level is close to equilibrium (lateral perturbation). We suggest that high maize yield crops are especially vulnerable to weather-related yield variations. These data support the belief that lower yields are more suitable for low-input conditions, because climate might be more severe in crops that interact strongly with productivity [14].

Our results also indicate that it may be important to consider explicitly the irrigation system and spatial variability. Rain-fed agriculture may be at risk as heat waves will be more intense, more frequent and longer (particularly in Seville, Cádiz, Almería, Navarra and Ávila; see Fig. 2). Irrigation seems to allow some tolerance to warming but future levels of water availability would be compromised if water restrictions and irrigation costs increased, as climate change projections indicate. We propose a new framework to estimate yield potential as the equilibrium yield or yield carrying capacity. Climate change is not uniform over Spain and the effectiveness of irrigated and rain-fed management varies with the location, producing different regional vulnerabilities and potential yields. Accordingly, the general strategies for adapting maize productivity to climate change will vary between different zones in Spain.

## Supporting Information

Figure S1 **Time series of maize yield level for rain-fed (red) and irrigated (blue) systems.** Each provinces of Spain were analyzed for 1996–2009.
(TIFF)

Figure S2 **Partial rate correlation function (** ***PRCF*** **).**
(TIFF)

Figure S3 **Comparison of observed crop yield levels (points,** ***obs*** **) for the period 1997–2009 with stochastic predictions from models fitted to the data until the year 1996 (broken line,** ***sim*** **) and 95% confidence intervals for forecasts (shaded area,** ***95PPU*** **).** P-factor is the percent of observations that are within the given uncertainty bounds and R-factor represents the average width of the given uncertainty bounds divided by the standard deviation of the observations. See Table S1 for description of models and variables.
(TIFF)

Figure S4 ***R*** **-functions: yield rate of change against the log observed yield level (with one year of delay).** Climate factors had vertical (additive) and lateral (non-additive) perturbations on the *R*-function. Colors indicate the value of the *R*-function. See Table S1 for description of models and variables.
(TIFF)

Figure S5 **A non-parametric approach to inference on Moran's** ***I*** **using 999 simulations (Monte Carlo permutation test).**
(TIFF)

Table S1 Summary statistics of nonlinear logistic models, 1996–2009. We evaluated pure Endogenous models (E), and additive (or Lateral, L) and non-additive (or Vertical, V) models that also represent the effect of exogenous perturbations. Different crop management systems were analyzed (*IR* =  irrigated and *RF* = rain-fed). *%Total* percentage of total crop production in Spain, *K* carrying capacity or potential yield, *rmax* maximum finite reproductive rate, *a* non-linearity coefficient, *c* the ratio between demand and offer of limiting resources, *b* coefficients for different exogenous effects, *R^2^* pseudo-coefficient of determination, *logLIK* log-likelihood, *RMSE* root-mean-square error and *AICc* corrected Akaike information criterion. NOTE: *p<0.05, **p<0.01, Number of not avaiable data (NA) were indicated by I. *CO2* carbon dioxide emission (*kt*, country-level emissions), and summary statistics of the growing season weather: *EMNT* extreme minimum temperature (°C), *EMXT* extreme maximum temperature (°C), *MMNT* mean minimum temperature (°C), *MMXT* mean maximum temperature (°C), *MNTM* mean temperature (°C), *EMXP* extreme maximum daily precipitation total (l/m2), *TPCP* total precipitation (l/m2).
(DOC)
