# Supplementary figures and images for: Spatio-Temporal Dynamics of Maize Yield Water Constraints under Climate Change in Spain
Source: PLoS One. 2014 May 30;9(5):e98220. doi: 10.1371/journal.pone.0098220 (PMC4039498; doi:10.1371/journal.pone.0098220)

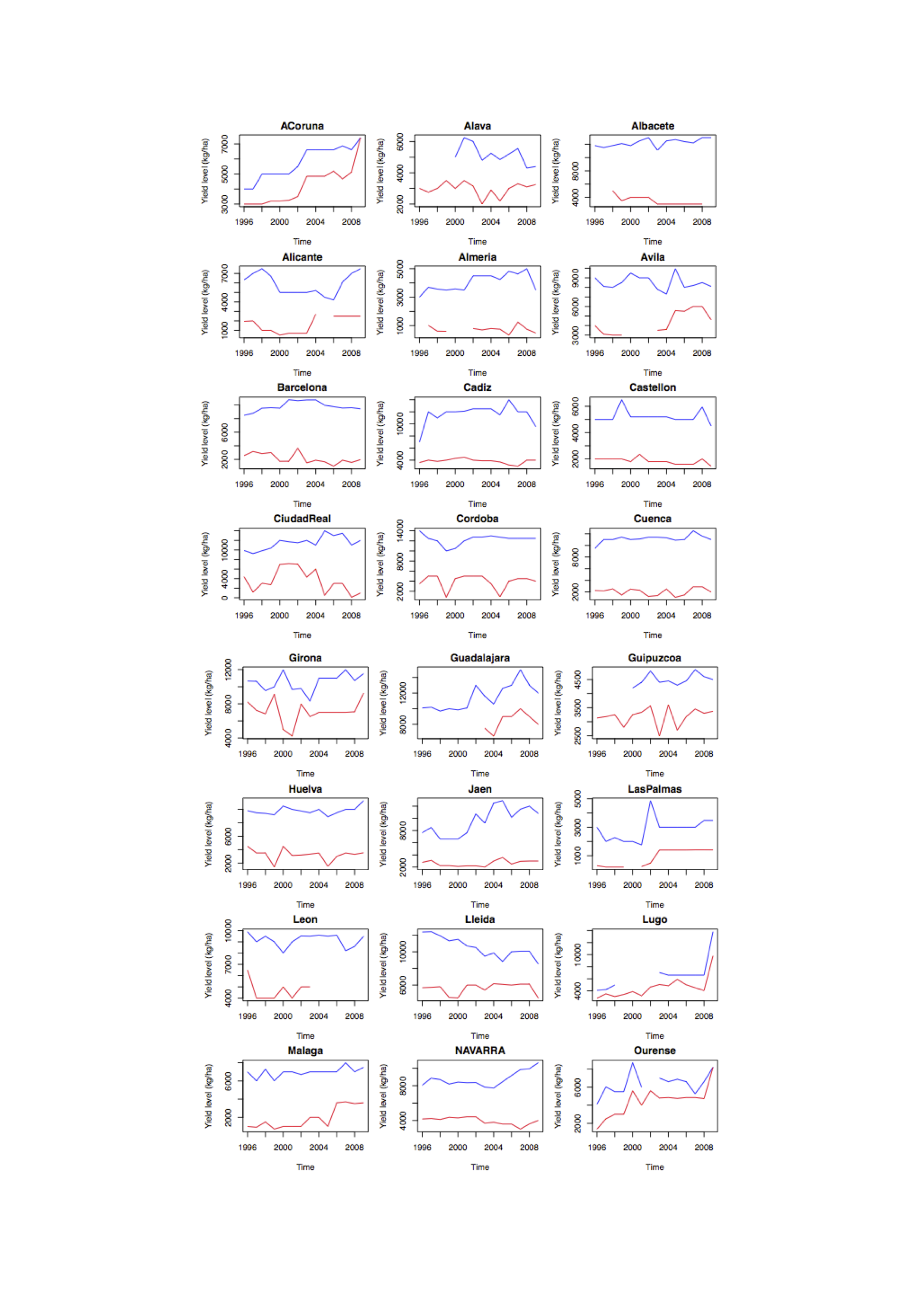

Supplement: Figure S1 — Time series of maize yield level for rain-fed (red) and irrigated (blue) systems. Each provinces of Spain were analyzed for 1996–2009. (TIFF) [file pone.0098220.s001.tiff]

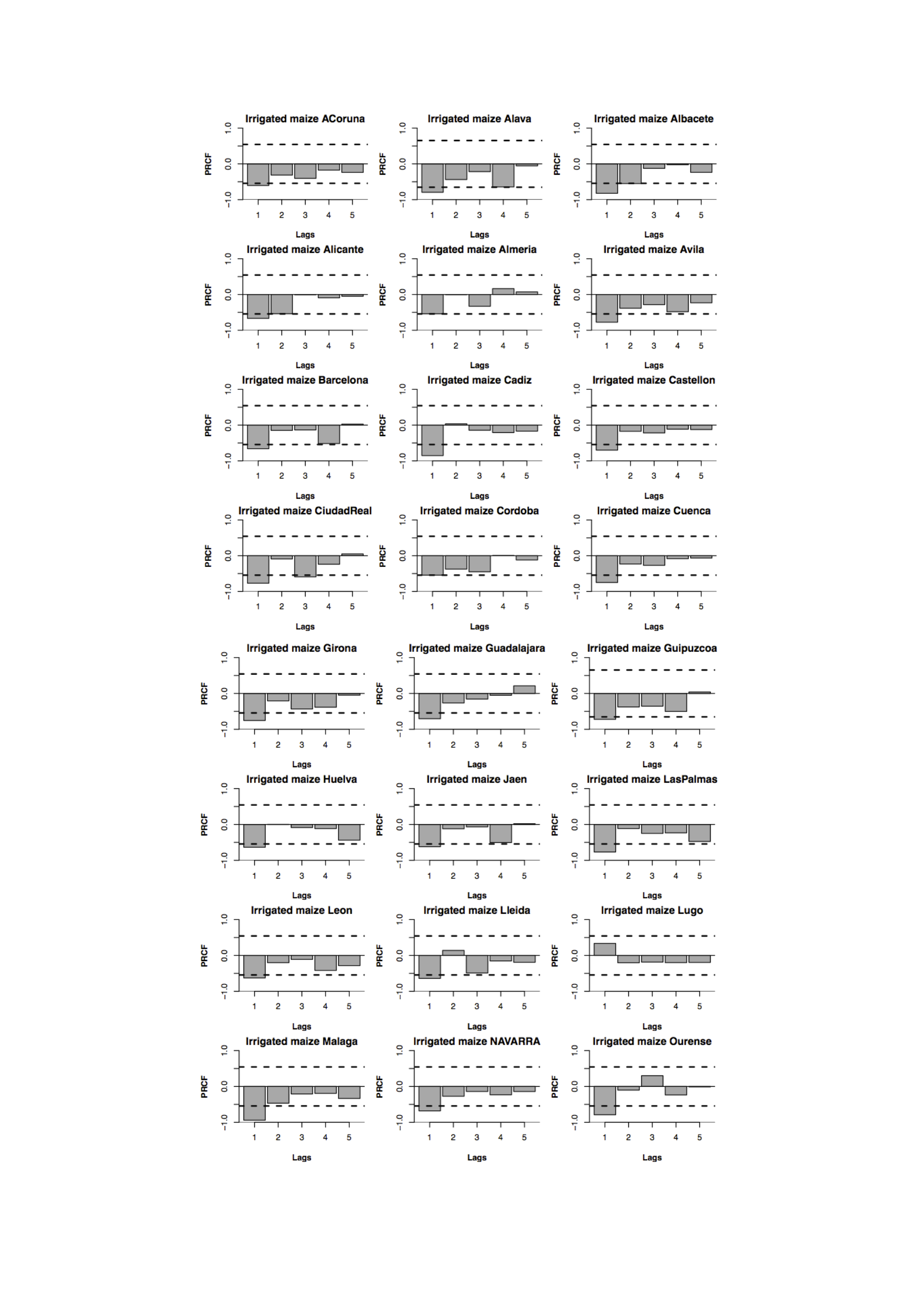

Supplement: Figure S2 — Partial rate correlation function ( PRCF ). (TIFF) [file pone.0098220.s002.tiff]

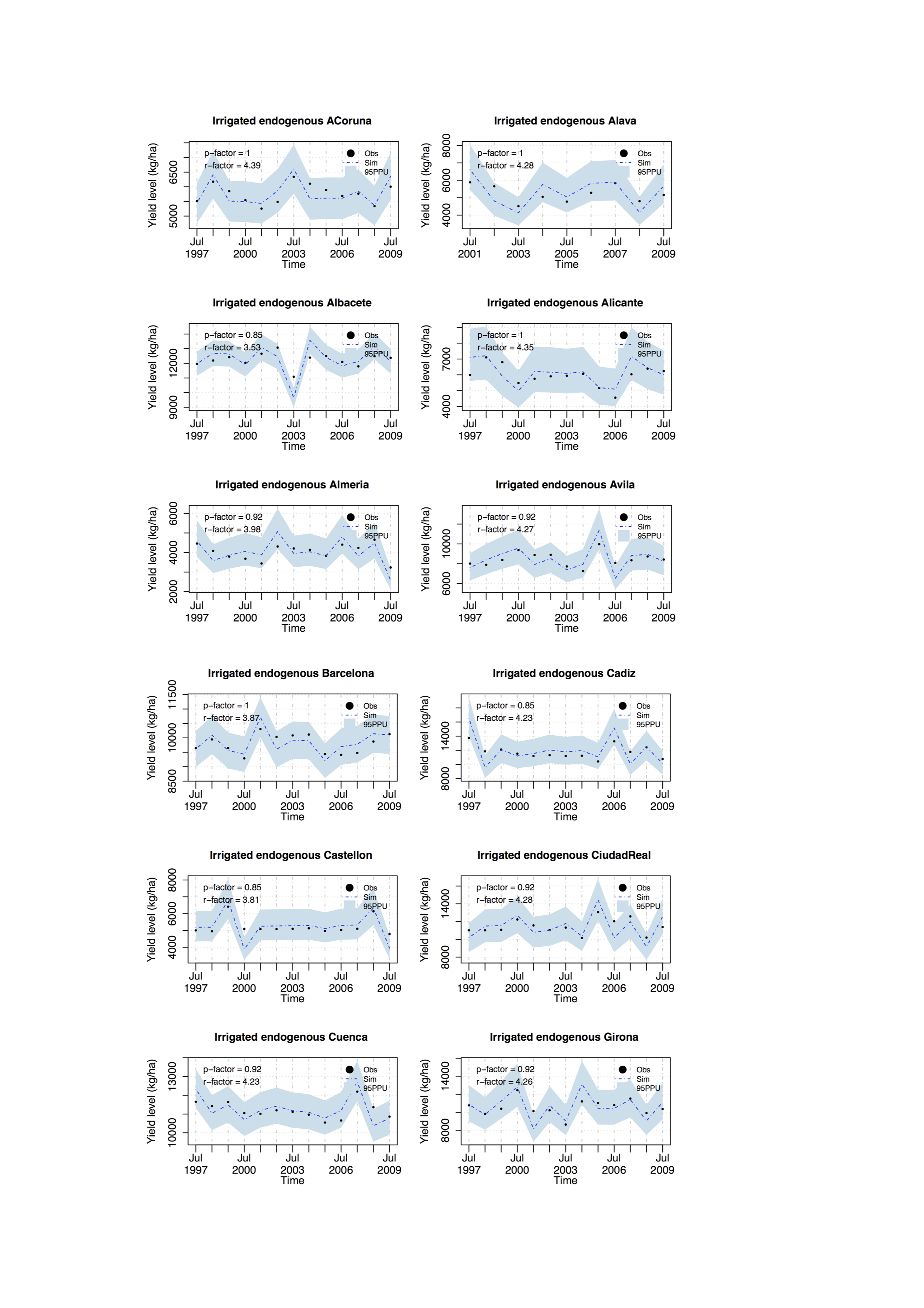

Supplement: Figure S3 — Comparison of observed crop yield levels (points, obs ) for the period 1997–2009 with stochastic predictions from models fitted to the data until the year 1996 (broken line, sim ) and 95% confidence intervals for forecasts (shaded area, 95PPU ). P-factor is the percent of observations that are within the given uncertainty bounds and R-factor represents the average width of the given uncertainty bounds divided by the standard deviation of the observations. See Table S1 for description of models and variables. (TIFF) [file pone.0098220.s003.tiff]

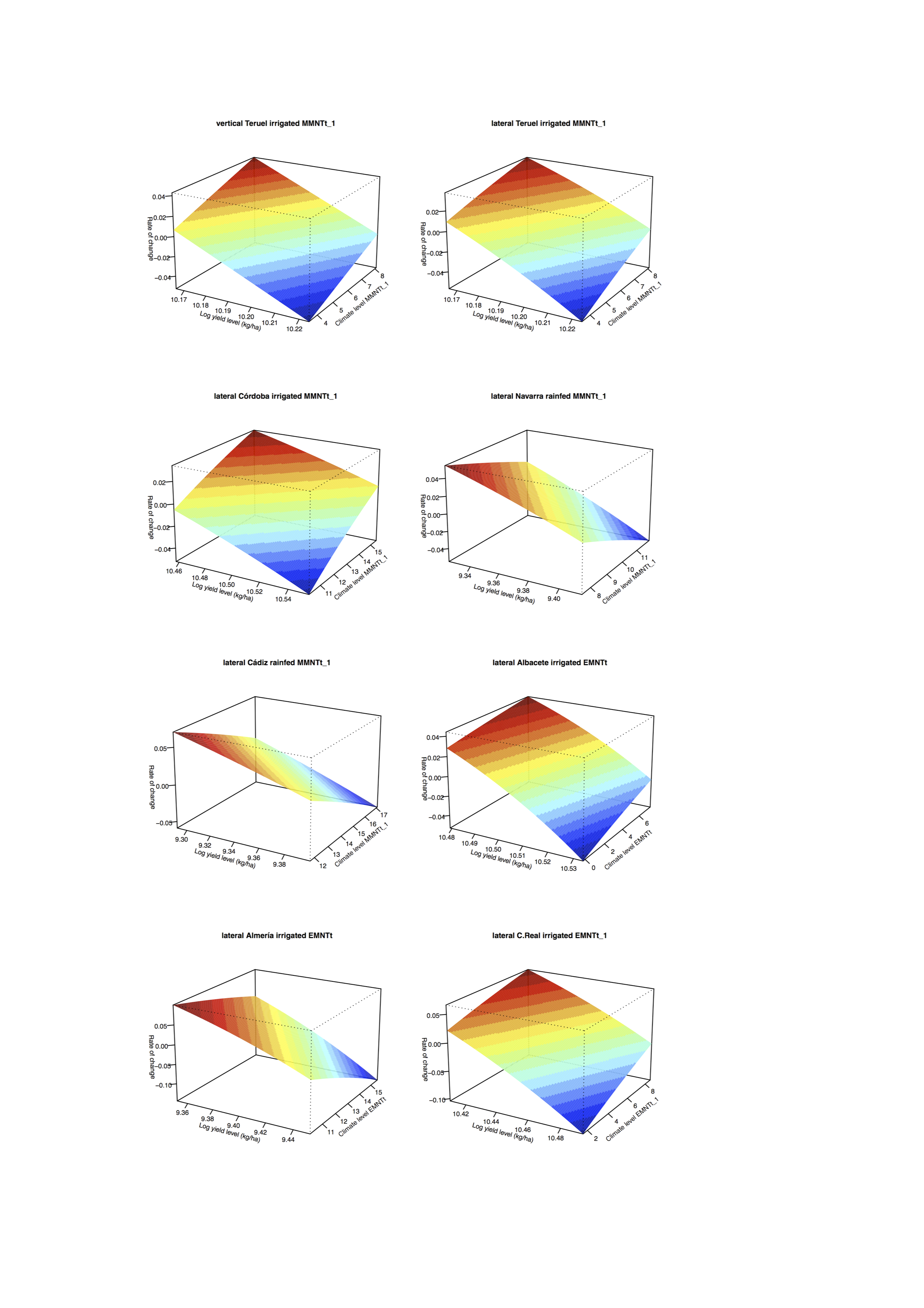

Supplement: Figure S4 — R -functions: yield rate of change against the log observed yield level (with one year of delay). Climate factors had vertical (additive) and lateral (non-additive) perturbations on the R-function. Colors indicate the value of the R-function. See Table S1 for description of models and variables. (TIFF) [file pone.0098220.s004.tiff]

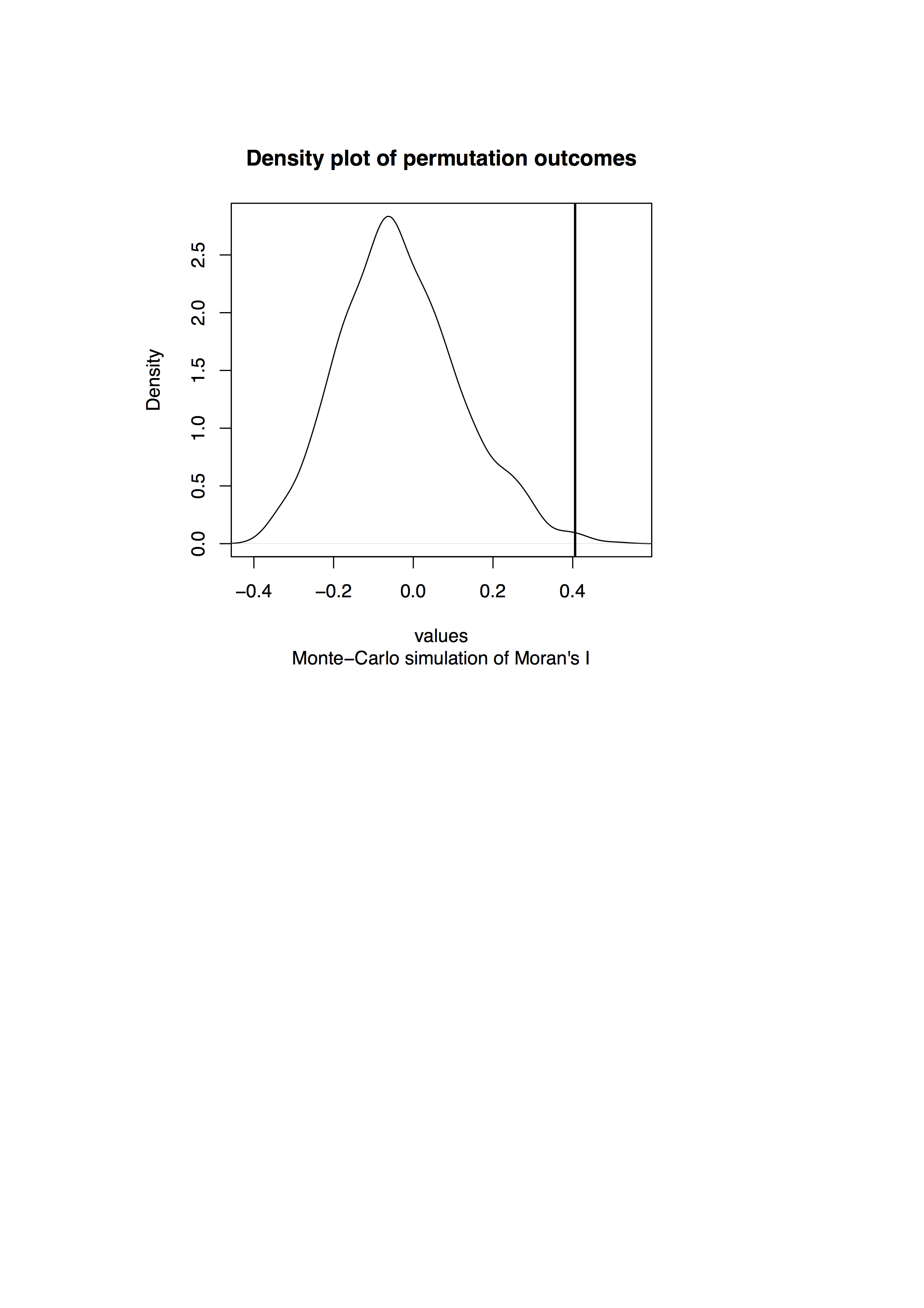

Supplement: Figure S5 — A non-parametric approach to inference on Moran's I using 999 simulations (Monte Carlo permutation test). (TIFF) [file pone.0098220.s005.tiff]
