# Supplementary material for: Spatio-Temporal Dynamics of Maize Yield Water Constraints under Climate Change in Spain
Source: PLoS One. 2014 May 30;9(5):e98220. doi: 10.1371/journal.pone.0098220 (PMC4039498; doi:10.1371/journal.pone.0098220)
Supplement: Table S1 — Summary statistics of nonlinear logistic models, 1996–2009. We evaluated pure Endogenous models (E), and additive (or Lateral, L) and non-additive (or Vertical, V) models that also represent the effect of exogenous perturbations. Different crop management systems were analyzed (IR = irrigated and RF = rain-fed). %Total percentage of total crop production in Spain, K carrying capacity or potential yield, rmax maximum finite reproductive rate, a non-linearity coefficient, c the ratio between demand and offer of limiting resources, b coefficients for different exogenous effects, R2 pseudo-coefficient of determination, logLIK log-likelihood, RMSE root-mean-square error and AICc corrected Akaike information criterion. NOTE: *p<0.05, **p<0.01, Number of not avaiable data (NA) were indicated by I. CO2 carbon dioxide emission (kt, country-level emissions), and summary statistics of the growing season weather: EMNT extreme minimum temperature (°C), EMXT extreme maximum temperature (°C), MMNT mean minimum temperature (°C), MMXT mean maximum temperature (°C), MNTM mean temperature (°C), EMXP extreme maximum daily precipitation total (l/m2), TPCP total precipitation (l/m2). (DOC) [file pone.0098220.s006.doc]

| ***%Tot*** | ***Sitio*** | ***Model*** | ***Syst*** | ***Climate*** | ***K*** | ***rmax*** | ***logLIK*** | ***R2*** | ***AICc*** | ***RMSE*** | ***a*** | ***b*** | ***c*** |
| --- | --- | --- | --- | --- | --- | --- | --- | --- | --- | --- | --- | --- | --- |
| 14,78 | León | V | IR | MMXTt | 23.89 | 0,12 | 38,11 | **0,75** | **-65,83** | 0,01 | 0,13* | 1,50** | 0,69 |
| 14,78 | León | L | IR | MMXTt | 27.70 | 0,12 | 38,07 | **0,75** | **-65,73** | 0,01 | 7,88** | -12,07** | -24,20 |
| 14,78 | León | E | IR |  | 27.41 | 0,12 | 32,76 | 0,44 | -58,44 | 0,02 | 7,63* |  | -80,09* |
| 14,78 | León 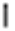7 | E | RF |  | 14.03 | 0,22 | 13,74 | **0,75** | **-20,38** | 0,03 | 5,93* |  | -58,14* |
| 6,53 | Lleida | E | IR |  | 32.01 | 0,13 | 35,34 | **0,50** | **-63,59** | 0,02 | 8,88* |  | -94,16* |
| 6,53 | Lleida | E | RF |  | 16.66 | 0,30 | 24,42 | **0,35** | **-41,75** | 0,04 | 2,95* |  | -29,88* |
| 6,32 | Zaragoza | E | IR |  | 26.84 | 0,39 | 24,88 | **0,42** | **-42,66** | 0,04 | 2,27* |  | -24,09* |
| 6,32 | Zaragoza | L | RF | TPCPt | 7.70 | 0,65 | 20,23 | **0,64** | **-30,06** | 0,05 | 0,66 | 0,38* | -8,36* |
| 6,32 | Zaragoza | E | RF |  | 7.26 | 0,65 | 16,27 | 0,33 | -25,44 | 0,07 | 1,01* |  | -9,41* |
| 5,10 | Albacete | L | IR | EMNTt | 36.76 | 0,12 | 42,84 | **0,81** | **-75,29** | 0,01 | 11,80** | -2,62* | -113,99** |
| 5,10 | Albacete | E | IR |  | 36.63 | 0,12 | 40,07 | 0,71 | -73,04 | 0,01 | 12,54** |  | -133,90** |
| 5,10 | Albacete 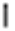4 | L | RF | EMNTt_1 | 10.50 | 0,13 | 25,47 | **0,86** | **-40,54** | 0,02 | 10,70** | -8,81* | -60,19* |
| 5,10 | Albacete 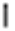4 | E | RF |  | 10.54 | 0,13 | 21,34 | 0,69 | -35,59 | 0,03 | 10,30** |  | -97,45** |
| 5,06 | Zamora | E | IR |  | 27.82 | 0,16 | 32,76 | **0,61** | **-58,43** | 0,02 | 7,57** |  | -79,30** |
| 5,06 | Zamora | E | RF |  | 7.26 | 0,85 | 16,23 | **0,59** | **-25,36** | 0,07 | 1,22** |  | -11,76** |
| 4,32 | Sevilla | L | IR | EMXTt_1 | 34.41 | 0,15 | 32,79 | **0,69** | **-55,19** | 0,02 | 5,44* | -9,41* | -12,07 |
| 4,32 | Sevilla | E | IR |  | 34.11 | 0,15 | 29,96 | 0,52 | -52,84 | 0,02 | 6,80* |  | -72,87* |
| 4,32 | Sevilla | L | RF | EMNTt | 7.01 | 2,16 | 13,12 | **0,82** | **-15,83** | 0,09 | 0,60** | 2,39* | -15,79** |
| 4,32 | Sevilla | E | RF |  | 7.70 | 2,16 | 8,00 | 0,60 | -8,91 | 0,13 | 0,58** |  | -4,42* |
| 4,27 | Toledo | E | IR |  | 33.58 | 0,27 | 33,72 | **0,63** | **-60,35** | 0,02 | 4,77** |  | -51,02** |
| 4,27 | Toledo 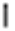2 | E | RF |  | 7.39 | 0,50 | 24,31 | **0,91** | **-41,52** | 0,03 | 3,50** |  | -31,87** |
| 3,43 | Navarra | V | IR | EMXPt_1 | 26.67 | 0,09 | 42,85 | **0,71** | **-75,30** | 0,01 | 1,90* | 0,07* | -20,20* |
| 3,43 | Navarra | L | IR | EMXPt_1 | 26.13 | 0,09 | 42,36 | **0,69** | **-74,32** | 0,01 | 8,79** | -0,73* | -88,23** |
| 3,43 | Navarra | E | IR |  | 26.10 | 0,09 | 38,76 | 0,46 | -70,43 | 0,01 | 8,56* |  | -89,46* |
| 3,43 | Navarra | L | RF | MMNTt_1 | 12.03 | 0,18 | 32,26 | **0,54** | **-54,13** | 0,02 | 3,81* | 6,61* | -68,45* |
| 2,32 | Girona | L | IR | MMXTt_1 | 10.56 | 0,28 | 31,15 | **0,73** | **-51,89** | 0,02 | 3,74** | -4,70* | -17,17 |
| 2,32 | Girona | V | IR | MNTMt_1 | 10.56 | 0,28 | 30,79 | **0,71** | **-51,19** | 0,02 | 0,09 | 2,41* | 1,56 |
| 2,32 | Girona | E | IR |  | 31.79 | 0,28 | 28,06 | 0,56 | -49,04 | 0,03 | 4,00** |  | -42,74** |
| 2,32 | Girona | E | RF |  | 20.97 | 0,64 | 19,97 | **0,62** | **-32,85** | 0,05 | 1,96** |  | -19,95** |
| 1,93 | Córdoba | L | IR | MMNTt_1 | 12.30 | 0,13 | 35,94 | **0,55** | **-61,48** | 0,02 | 3,04 | -7,02* | -0,74 |
| 1,93 | Córdoba | L | RF | TPCPt | 11.34 | 1,73 | 10,45 | **0,68** | **-10,50** | 0,11 | 0,62** | 0,15* | -6,06** |
| 1,93 | Córdoba | L | RF | EMXPt | 11.87 | 1,73 | 10,90 | **0,70** | **-11,41** | 0,10 | 0,66** | 0,37* | -7,47** |
| 1,93 | Córdoba | E | RF |  | 12.32 | 1,73 | 7,86 | 0,52 | -8,64 | 0,13 | 0,64* |  | -5,48* |
| 1,75 | Ciudad Real | V | IR | EMNTt_1 | 34.39 | 0,24 | 33,72 | **0,77** | **-57,04** | 0,02 | 0,35* | 0,79* | -2,29 |
| 1,75 | Ciudad Real | L | IR | EMNTt_1 | 34.63 | 0,24 | 33,19 | **0,75** | **-55,97** | 0,02 | 5,41** | -3,37* | -42,28** |
| 1,75 | Ciudad Real | E | IR |  | 34.42 | 0,24 | 30,03 | 0,59 | -52,97 | 0,02 | 4,71** |  | -50,63** |
| 1,75 | Ciudad Real | E | RF |  | 9.61 | 2,29 | 4,83 | **0,59** | **-2,57** | 0,17 | 0,52** |  | -3,94* |
| 1,43 | Guadalajara | E | IR |  | 34.37 | 0,25 | 27,24 | **0,43** | **-47,40** | 0,03 | 3,56* |  | -38,57* |
| 1,43 | Guadalajara 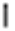8 | E | RF |  | 25.20 | 0,33 | 14,77 | **0,82** | **-22,45** | 0,02 | 4,99* |  | -51,68* |
| 1,38 | A Coruña | E | IR |  | 17.06 | 0,22 | 33,34 | **0,44** | **-59,60** | 0,02 | 3,97* |  | -40,20* |
| 1,38 | A Coruña | E | RF |  | 12.85 | 0,37 | 23,11 | **0,36** | **-39,13** | 0,04 | 2,40* |  | -23,70* |
| 1,13 | Teruel | V | IR | MMNTt_1 | 21.33 | 0,15 | 37,30 | **0,77** | **-64,20** | 0,01 | 0,26 | 0,83* | -1,20 |
| 1,13 | Teruel | L | IR | MMNTt_1 | 26.55 | 0,15 | 37,07 | **0,76** | **-63,73** | 0,01 | 6,58** | -4,96* | -45,79* |
| 1,13 | Teruel | V | IR | MNTMt_1 | 25.70 | 0,15 | 37,36 | **0,77** | **-64,32** | 0,01 | 0,08 | 2,45* | 1,66 |
| 1,13 | Teruel | E | IR |  | 26.56 | 0,15 | 34,08 | 0,62 | -61,07 | 0,02 | 7,28** |  | -76,06** |
| 1,13 | Teruel | V | RF | TPCPt_1 | 8.54 | 1,74 | 8,92 | **0,72** | **-7,44** | 0,12 | 0,26** | 0,63* | -0,71 |
| 1,13 | Teruel | L | RF | TPCPt_1 | 9.20 | 1,74 | 8,94 | **0,72** | **-7,48** | 0,12 | 0,79** | -0,37* | -4,64* |
| 1,13 | Teruel | V | RF | EMXPt | 6.29 | 1,74 | 8,88 | **0,72** | **-7,36** | 0,12 | 0,11 | 1,29* | 1,12 |
| 1,13 | Teruel | L | RF | EMXPt | 9.43 | 1,74 | 9,27 | **0,74** | **-8,15** | 0,12 | 0,49* | -0,84* | 0,16 |
| 1,13 | Teruel | E | RF |  | 9.43 | 1,74 | 5,73 | 0,55 | -4,37 | 0,16 | 0,62** |  | -5,12* |
| 1,00 | Palencia | E | IR |  | 22.22 | 0,55 | 17,25 | **0,39** | **-27,40** | 0,06 | 1,40* |  | -14,61* |
| 1,00 | Palencia 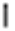4 | E | RF |  | 18.15 | 0,86 | 9,53 | **0,59** | **-11,98** | 0,09 | 1,45* |  | -14,37* |
| 0,94 | Cádiz | E | IR |  | 35.62 | 0,54 | 29,61 | **0,71** | **-52,14** | 0,02 | 2,40** |  | -25,77** |
| 0,94 | Cádiz | L | RF | MMNTt_1 | 11.55 | 0,29 | 31,00 | **0,57** | **-51,61** | 0,02 | 2,00* | 5,27* | -44,94* |
| 0,94 | Cádiz | L | RF | MNTMt_1 | 11.83 | 0,29 | 31,82 | **0,62** | **-53,23** | 0,02 | 2,90* | 14,53* | -98,35* |
| 0,89 | Pontevedra | E | IR |  | 16.95 | 0,20 | 27,60 | **0,34** | **-48,12** | 0,03 | 4,20* |  | -42,51* |
| 0,89 | Pontevedra | E | RF |  | 18.15 | 0,33 | 27,74 | **0,36** | **-48,39** | 0,03 | 2,17* |  | -21,47* |
| 0,37 | Cuenca | E | IR |  | 33.41 | 0,15 | 37,91 | **0,54** | **-68,72** | 0,01 | 6,73** |  | -72,00** |
| 0,37 | Cuenca | E | RF |  | 6.28 | 0,66 | 13,35 | **0,61** | **-19,60** | 0,09 | 1,91** |  | -17,12** |
| 0,35 | Ourense | L | IR | MNTMt_1 | 19.34 | 0,46 | 24,36 | **0,75** | **-38,33** | 0,04 | 2,87** | 12,02* | -86,68** |
| 0,35 | Ourense | E | IR |  | 19.04 | 0,46 | 21,11 | 0,58 | -35,12 | 0,05 | 2,52** |  | -25,61** |
| 0,35 | Ourense | V | RF | CO2t | NA | 0,62 | 18,23 | **0,72** | **-26,06** | 0,05 | 12,72 | -0,05** | -124,07 |
| 0,35 | Ourense | E | RF |  | 13.26 | 0,62 | 15,95 | 0,46 | -24,81 | 0,07 | 1,75* |  | -17,09* |
| 0,31 | Jaén | E | IR |  | 28.17 | 0,34 | 21,36 | **0,38** | **-35,64** | 0,05 | 2,20* |  | -23,62* |
| 0,31 | Jaén | E | RF |  | 8.02 | 0,41 | 21,05 | **0,44** | **-35,01** | 0,05 | 2,09* |  | -19,68* |
| 0,29 | Lugo | L | IR | CO2t | 22.26 | 0,74 | 36,24 | **0,98** | **-62,07** | 0,01 | -0,18 | 0,28** | -2,04 |
| 0,29 | Lugo | E | RF |  | 13.58 | 0,88 | 13,15 | **0,45** | **-19,20** | 0,09 | 1,38* |  | -13,26* |
| 0,23 | Barcelona | E | IR |  | 29.48 | 0,12 | 40,44 | **0,39** | **-73,78** | 0,01 | 6,86* |  | -72,72* |
| 0,23 | Barcelona | E | RF |  | 6.48 | 0,75 | 14,20 | **0,68** | **-21,30** | 0,08 | 1,69** |  | -15,12** |
| 0,20 | Ávila | E | IR |  | 25.50 | 0,31 | 28,54 | **0,59** | **-49,99** | 0,03 | 3,68** |  | -38,51** |
| 0,20 | Ávila | V | RF | CO2t | 13.46 | 0,44 | 25,39 | **0,78** | **-40,38** | 0,03 | 0,18* | 0,25* | -0,43 |
| 0,20 | Ávila | L | RF | CO2t | 12.91 | 0,44 | 25,13 | **0,77** | **-39,85** | 0,03 | 1,42* | -0,53* | -7,56 |
| 0,13 | Soria | E | IR |  | 29.81 | 0,11 | 36,61 | **0,67** | **-66,12** | 0,01 | 11,91** |  | -124,91** |
| 0,13 | Soria | L | RF | TPCPt_1 | 26.15 | 0,19 | 32,14 | **0,84** | **-53,88** | 0,02 | 7,98** | -0,37* | -80,76** |
| 0,13 | Soria | L | RF | EMXPt_1 | 26.10 | 0,19 | 32,40 | **0,84** | **-54,40** | 0,02 | 7,17** | -1,21* | -68,69** |
| 0,13 | Soria | E | RF |  | 26.18 | 0,19 | 28,94 | 0,73 | -50,79 | 0,03 | 7,82** |  | -81,21** |
| 0,11 | Málaga | E | IR |  | 20.97 | 0,20 | 38,14 | **0,88** | **-69,19** | 0,01 | 8,75** |  | -88,68** |
| 0,11 | Málaga | V | RF | EMNTt_1 | 8.45 | 1,28 | 16,05 | **0,79** | **-21,70** | 0,07 | 0,07* | 3,45* | 2,23** |
| 0,11 | Málaga | L | RF | EMNTt_1 | 5.79 | 1,28 | 15,61 | **0,78** | **-20,81** | 0,07 | 1,01** | -2,80* | 4,67 |
| 0,11 | Málaga | E | RF |  | 5.47 | 1,28 | 11,47 | 0,58 | -15,84 | 0,10 | 0,95** |  | -7,93** |
| 0,11 | Valencia | E | IR |  | 23.28 | 0,46 | 22,77 | **0,38** | **-38,45** | 0,04 | 1,69* |  | -17,77* |
| 0,11 | Valencia 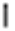5 | E | RF |  | 3.50 | 0,11 | 16,37 | **0,54** | **-25,65** | 0,04 | 9,61* |  | -80,63* |
| 0,05 | Huelva | E | IR |  | 29.29 | 0,13 | 34,55 | **0,39** | **-62,00** | 0,02 | 6,60* |  | -69,92* |
| 0,05 | Huelva | E | RF |  | 9.41 | 1,17 | 13,82 | **0,66** | **-20,54** | 0,08 | 1,18** |  | -10,64** |
| 0,04 | Alicante | E | IR |  | 17.79 | 0,37 | 25,24 | **0,44** | **-43,38** | 0,03 | 2,03* |  | -20,86* |
| 0,03 | Guipuzcoa 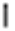5 | E | IR |  | 13.50 | 0,09 | 26,29 | **0,54** | **-45,48** | 0,01 | 11,43* |  | -111,11* |
| 0,03 | Guipuzcoa | E | RF |  | 9.65 | 0,37 | 29,32 | **0,81** | **-51,56** | 0,03 | 4,62** |  | -43,38** |
| 0,02 | Las Palmas | E | IR |  | 8.44 | 1,02 | 14,94 | **0,56** | **-22,78** | 0,08 | 0,99** |  | -8,93** |
| 0,02 | S.C. de Tenerife | E | RF |  | 4.51 | 0,29 | 26,38 | **0,59** | **-45,66** | 0,03 | 3,46** |  | -30,35** |
| 0,02 | Tarragona | E | IR |  | 24.39 | 0,20 | 33,02 | **0,48** | **-58,94** | 0,02 | 4,82* |  | -50,30* |
| 0,02 | Tarragona | E | RF |  | 9.65 | 0,69 | 14,14 | **0,35** | **-21,18** | 0,08 | 1,08* |  | -10,28* |
| 0,01 | Castellón | E | IR |  | 15.64 | 0,26 | 28,08 | **0,62** | **-49,07** | 0,03 | 4,31** |  | -42,97** |
| 0,01 | Castellón | E | RF |  | 5.51 | 0,27 | 26,90 | **0,65** | **-46,72** | 0,03 | 4,46** |  | -39,73** |
| 0,01 | Vizcaya | E | RF |  | 9.06 | 0,32 | 29,25 | **0,80** | **-51,41** | 0,03 | 5,10** |  | -47,61** |
| 0,00 | Álava 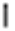5 | E | IR |  | 15.56 | 0,22 | 19,03 | **0,63** | **-30,98** | 0,03 | 5,33* |  | -52,96* |
| 0,00 | Álava | L | RF | MMXTt | 9.02 | 0,37 | 24,38 | **0,69** | **-38,35** | 0,04 | 3,27** | 6,14* | -60,51** |
| 0,00 | Álava | E | RF |  | 8.80 | 0,37 | 21,14 | 0,49 | -35,19 | 0,05 | 2,74* |  | -25,88* |
| 0,00 | Almería | L | IR | EMNTt | 12.17 | 0,25 | 31,87 | **0,78** | **-53,34** | 0,02 | 5,57** | 8,18** | -92,44** |
| 0,00 | Almería | E | IR |  | 12.22 | 0,25 | 26,15 | 0,48 | -45,21 | 0,03 | 4,73* |  | -45,90* |
| 0,00 | Almería 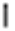2 | L | RF | EMNTt | 2.09 | 1,32 | 14,96 | **0,81** | **-19,53** | 0,07 | 1,08** | 3,98* | -26,79* |
| 0,00 | Almería 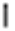2 | E | RF |  | 2.12 | 1,32 | 11,27 | 0,65 | -15,45 | 0,09 | 0,95** |  | -7,00** |
